# The Asian bush mosquito *Aedes japonicus japonicus* (Diptera: Culicidae) in Europe, 17 years after its first detection, with a focus on monitoring methods

**DOI:** 10.1186/s13071-019-3349-3

**Published:** 2019-03-14

**Authors:** Marcel B. Koban, Helge Kampen, Dorothee E. Scheuch, Linus Frueh, Cornelius Kuhlisch, Nele Janssen, Johannes L. M. Steidle, Günter A. Schaub, Doreen Werner

**Affiliations:** 1 Leibniz-Centre for Agricultural Landscape Research, Müncheberg, Germany; 2 University of Hohenheim, Stuttgart, Germany; 3 Friedrich-Loeffler-Institute, Federal Research Institute for Animal Health, Insel Riems, Greifswald, Germany; 4 Ruhr-University, Bochum, Germany

**Keywords:** *Aedes japonicus japonicus*, Asian bush mosquito, Asian rock pool mosquito, Europe, Spread, Invasive species, Monitoring, Surveillance

## Abstract

After the first detection of the Asian bush mosquito *Aedes japonicus japonicus* in the year 2000 in France, its invasive nature was revealed in 2008 in Switzerland and Germany. In the following years, accumulating reports have shown that *Ae. j. japonicus* succeeded in establishing in several European countries. Surveillance efforts suggest that there are currently four populations in Europe, with the largest one, formed by the recent fusion of several smaller populations, ranging from West Germany, with extensions to Luxembourg and French Alsace, southwards to Switzerland and continuing westwards through Liechtenstein to western Austria. This paper summarises the present distribution of *Ae. j. japonicus* in Europe, based on published literature and hitherto unpublished findings by the authors, and critically reviews the monitoring strategies applied. A proposal for a more standardised monitoring approach is provided, aiming at the harmonisation of future data collections for improving the comparability between studies and the suitability of collected data for further research purposes, e.g. predictive modelling approaches.

## Background

The Asian bush mosquito or Asian rock pool mosquito *Aedes* (*Hulecoeteomyia*) *japonicus japonicus* (Theobald, 1901) is a highly invasive culicid species originating from East Asia (Japan, Korea, southern China, southeastern Russia) [1]. Outside its native distribution area, it was first reported from New Zealand in 1993, where larvae of *Ae. j. japonicus* were found in used tyres imported from Japan [2]. Although additional introductions were reported until 2003 (no pertinent data are available from 2003 onwards), there is no evidence that this species has become established in New Zealand [3].

In contrast, the species has successfully invaded North America and is now considered established in 33 US states and parts of Canada [4–8], presumably following repeated importations with used tyres and subsequent continental spread since 1999 [9, 10].

In Europe, *Ae. j. japonicus* was first detected in 2000 [11] and has since emerged in numerous countries, either through continental spread or additional introduction events from overseas [6]. While the Asian tiger mosquito *Aedes* (*Stegomyia*) *albopictus* (Skuse, 1895) and the yellow fever mosquito *Aedes* (*Stegomyia*) *aegypti *(Linnaeus, 1762) were considered responsible for several disease outbreaks in Europe after their establishment [12], there are no confirmed reports thus far of pathogen transmission through *Ae. j. japonicus* in the field, although it is a competent vector of several disease agents in the laboratory [13–19]. In addition, the isolation of La Crosse, Cache Valley and West Nile viruses or their respective RNAs from field-collected adults and the detection of La Crosse virus RNA in *Ae. j. japonicus* eggs suggests a possible role as a vector [20–22]. Knowing its geographical distribution is therefore essential from both a public and an animal health point of view, although eradication from Europe is no longer considered possible [6].

Several methods are available to detect and track *Ae. j. japonicus* populations, targeting all life stages of the species. In addition to adult trapping and ovitrapping [23–26], the surveillance of larval habitats is an appropriate cost-effective method [27].

This contribution will update the distribution of *Ae. j. japonicus* in Europe by the end of the mosquito season 2017, provide an overview over the genetic relationship of European populations and review the methods used to monitor this invasive species. Finally, a more standardised monitoring approach is proposed, aiming at the harmonisation of future data collections for improving the comparability between studies and the suitability of collected data for further research purposes, e.g. predictive modelling approaches.

## Methods

### Criteria for inclusion of reports

Articles, abstracts and presentations were analysed if findings of *Ae. j. japonicus* in previously non-infested areas in Europe were presented. In these cases, studies carried out until December 2017 were included. Studies on methodological evaluations conducted in areas already known to be infested were not considered.

### Sources

To find pertinent studies, PubMed, Google Scholar and Web of Science were the main search engines for the terms “*Aedes japonicus*”, “*Ochlerotatus japonicus*”, “*Ae. japonicus*”, “*Oc. japonicus*” and “*Aedes*”. In addition, the working group’s reference collection was searched for relevant information. The search results were manually scanned for studies concerning findings in new geographical areas of European countries.

### Data extraction

Coordinates provided for *Ae. j. japonicus* collection sites were copied to a CSV file and imported as a layer in QGIS. If GPS coordinates of collection sites were not provided in a study, data points were extracted by overlaying the included maps, using the “GDAL Georeferencer plugin” for QGIS and marking the dots manually. Further data extraction, e.g. collection dates or periods were extracted manually and collected in a separate CSV file.

## Detection, spread and current distribution of *Ae. j. japonicus* in Europe

After the first detection of *Ae. j. japonicus* in Europe (northwestern France [11]), observations have been published from numerous European countries suggesting continuous importation, e.g. through the used tyre trade, or quick dispersal of the species.

Despite two guidelines on mosquito surveillance, published by the ECDC [28, 29] and aiming at standardisation, monitoring efforts in Europe show a wide variation of methodological approaches. Differences can be found in the trigger of monitoring efforts, life stages targeted, traps used, structures searched, size of the area monitored and annual frequency of monitoring activities (Table 1). This section reviews the approaches and circumstances of initial local or regional findings of *Ae. j. japonicus* in Europe. Each subsection refers to originally detected populations and their subsequent development, ending in the delineation of the current populations in Europe.

**Table 1:** Specifics of the reviewed studies, including monitoring trigger, scope and methods used. A “dynamically adjusted” area is given if monitoring efforts were expanded while the field work was conducted. In the column “Year of first detection”, if the presence of *Ae. j. japonicus* in the monitored country was already reported, the corresponding study is referenced

Monitored country	Year of first detection	Trigger of monitoring	Monitoring area	Monitoring scope	Monitoring locations	Monitoring methods	No. of visits at the same sites	Population size (km^2^)	Additional information	Reference
France	2000	Passive monitoring	Fixed	Local	Tyre trading company	Larval sampling, adult trapping	6	Not stated	Eradicated [41]	[11]
Switzerland, Germany, France	2008 Switzerland, Germany	Submitted specimen	Dynamically adjusted	Regional	Cemeteries	Larval sampling	1	1400		[30]
Germany	2008 [30]	Report [30]	Not stated	Regional	Cemeteries	Larval sampling	2 (1 per year)	2200	Monitoring area expanded in second year	[31]
Germany	2008 [30]	Not stated	Fixed	Regional	Cemeteries, camping site	Larval sampling	1	Not stated		[32]
Germany	2008 [30]	Report [30, 31]	Fixed	Local	Along motorways	Adult trapping	8 (1 per week)	Not stated		[33]
Germany	2008 [30]	Report [31]	Fixed	Regional (federal state)	Cemeteries (mainly)	Larval sampling	1	1200 and 4000	Grid overlay, cell size: 135 km^2^; sites of [31] were included	[34]
Germany	2008 [30]	Report [34]	Fixed	Regional (federal state)	Cemeteries (mainly)	Larval sampling	1	Not stated	Revisited locations from [34]	[35]
France	2013	Report [30, 31]	Fixed	Regional	Cemeteries, others	Larval sampling, adult trapping	Multiple (not further specified)	Not stated		[36]
Austria, Germany, Hungary, Liechtenstein, Switzerland	2012 Hungary; 2015 Liechtenstein	Report [30, 31, 49]	Dynamically adjusted	Regional	Human settlements, forests, cemeteries	Ovitraps, larval sampling	Multiple (not further specified)	Not stated		[37]
Belgium	2002	Passive monitoring	Fixed	Local	Tyre trading company	Hand catching (human bait), larval sampling, adult trapping	Multiple (not further specified)	Not stated	No evidence for spread of population	[39]
Belgium	2002 [39]	Passive monitoring	Fixed	Local	Tyre trading company	Larval sampling, adult trapping	Multiple (not further specified)	Not stated	No evidence for spread of population; eradicated [41]	[40]
Germany	2008 [30]	Citizen science project “Mueckenatlas”	Dynamically adjusted	Regional	Cemeteries, private garden	Larval sampling	1	2000		[42]
Germany	2008 [30]	Report [42]	Dynamically adjusted	Regional	Cemeteries	Larval sampling	Multiple (1 per year)	8900	Grid overlay, cell size: 100 km^2^	[44, 60]
Germany	2008 [30]	Citizen science project “Mueckenatlas”	Dynamically adjusted	Regional	Cemeteries	Larval sampling	1	–	Only central water basins inspected	[45]
Germany	2008 [30]	Report [45]	Dynamically adjusted	Regional	Cemeteries	Larval sampling	Multiple (1 per year)	800	Grid overlay, cell size: 100 km^2^	[44, 60]
The Netherlands	2012	National framework for mosquito surveys	Dynamically adjusted	Local	Tyre trading company, allotment gardens, cemeteries, forests	Larval sampling, adult trapping	Multiple (not further specified)	Not stated	Population reduced but not eradicated [48]	[47]
Austria, Slovenia	2011	Not stated	Not stated	Local	“Kneipp” site, human settlements	Larval sampling	1	Not stated		[49]
Austria, Germany, Hungary, Liechtenstein, Switzerland	2012 Hungary, 2015 Liechtenstein	Report [30, 31, 49]	Dynamically adjusted	Regional	Human settlements, forests, cemeteries	Ovitraps, larval sampling	Multiple (not further specified)	Not stated		[37]
Austria, Italy	2015 Italy	Report [49]	Dynamically adjusted	Regional	Human settlements, forests, cemeteries	Larval sampling	–	Not stated		[50]
Slovenia	2011 [49]	Report [49]	Fixed	National	Human settlements, cemeteries, tire trading company	Larval sampling	Multiple (2 per year)	Not stated		[52]
Croatia	2013	Unknown	Fixed	Regional	Urban areas, cemeteries	Ovitraps, larval sampling	Multiple (not further specified)	Not stated		[53]
Austria, Germany	2008 Germany [30]; 2011 Austria [49]	Citizen science project “Mueckenatlas”	Dynamically adjusted	Regional	Cemeteries, private garden	Larval sampling	1	900	Grid overlay, cell size: 100 km^2^	[54]

### France/Switzerland/southwestern Germany/Liechtenstein/western Austria

In 2000, two larvae of *Ae. j. japonicus* were found in Normandy, northwestern France, in used tyres [11]. The larvae and breeding source were successfully eliminated [30].

Schaffner et al. [30] reported the first finding of *Ae. j. japonicus* in northern Switzerland and southwestern Germany in July 2008. Monitoring activities included the inspection of almost 3550 possible breeding habitats in Switzerland and bordering Germany and France. A source of introduction of this population was not identified [30].

Following this first detection of *Ae. j. japonicus* in Germany, Becker et al. [31] started a monitoring programme in southwestern Germany in 2009 to check for further distribution. Flower vases in cemeteries, used tyres and other water-holding containers in 86 villages were examined, and an infested area of approximately 2200 km^2^ was found. Locations were chosen due to their proximity to the Swiss border and the infested areas described by Schaffner et al. [30]. In 2010, the surveyed area was extended to 155 municipalities (villages visited in 2009 included) to account for the already well-established population.

Shortly thereafter, Schneider [32] found immature stages of *Ae. j. japonicus* in water-holding containers, e.g. vases, stone basins and rain barrels, in four cemeteries (of five inspected) and on one camping site in 2011. The southernmost site was located 80 km north of the *Ae. j. japonicus* distribution area previously reported by Becker et al. [31]. It was speculated that the species had reached the studied area by passive transportation rather than by active expansion [32] since it was thought to have a low dispersal range [9]. Interestingly, the northernmost location examined was about 10 km south of the airport of Stuttgart, which, according to Schneider [32], could be a possible introduction site, although no evidence exists of *Ae. j. japonicus* introductions via airports [30].

Coincidentally to the findings of larvae by Schneider [32], Werner et al. [33] reported the first trapping of adult females in southwestern Germany in the summer of 2011.

The studies of Schneider [32] and Werner et al. [33] led to the expansion of the monitoring activities in South Germany, with Huber et al. [34] conducting field investigations encompassing the entire federal state of Baden-Wuerttemberg (almost 35,000 km^2^) in 2011. Remarkably, the results suggested that the distribution area near the border to Switzerland had decreased, but at the same time, another, much larger infested area was discovered between the city of Stuttgart and the Swabian Mountains [34]. One year later, in 2012, Huber et al. [35] revisited the previously inspected sites and registered an increase from 54 to 124 positive municipalities. Genetic analyses indicated that the new population was likely to be the result of a northward spread of the southwestern population [35].

In 2013, Krebs et al. [36] found *Ae. j. japonicus* larvae only in one of nine deliberately selected cemeteries in French Alsace. Yet, immature stages were collected from additional random locations in a 6 km radius around the positive cemetery. Krebs et al. [36] concluded that the Swiss/German population had expanded to France, declared the establishment of *Ae. j. japonicus* in France, and considered eradication on the French territory unrealistic.

In 2011, Seidel et al. [37] investigated natural and artificial *Ae. j. japonicus* breeding sites in settlements and forests in western Austria. The study was expanded once developmental stages were detected in April 2015, resulting in a substantial increase in the survey area coverage. Eventually, the Asian bush mosquito was not only found in parts of Austria but also in Liechtenstein, Switzerland and southwestern Germany [37].

**Fig. 1:**
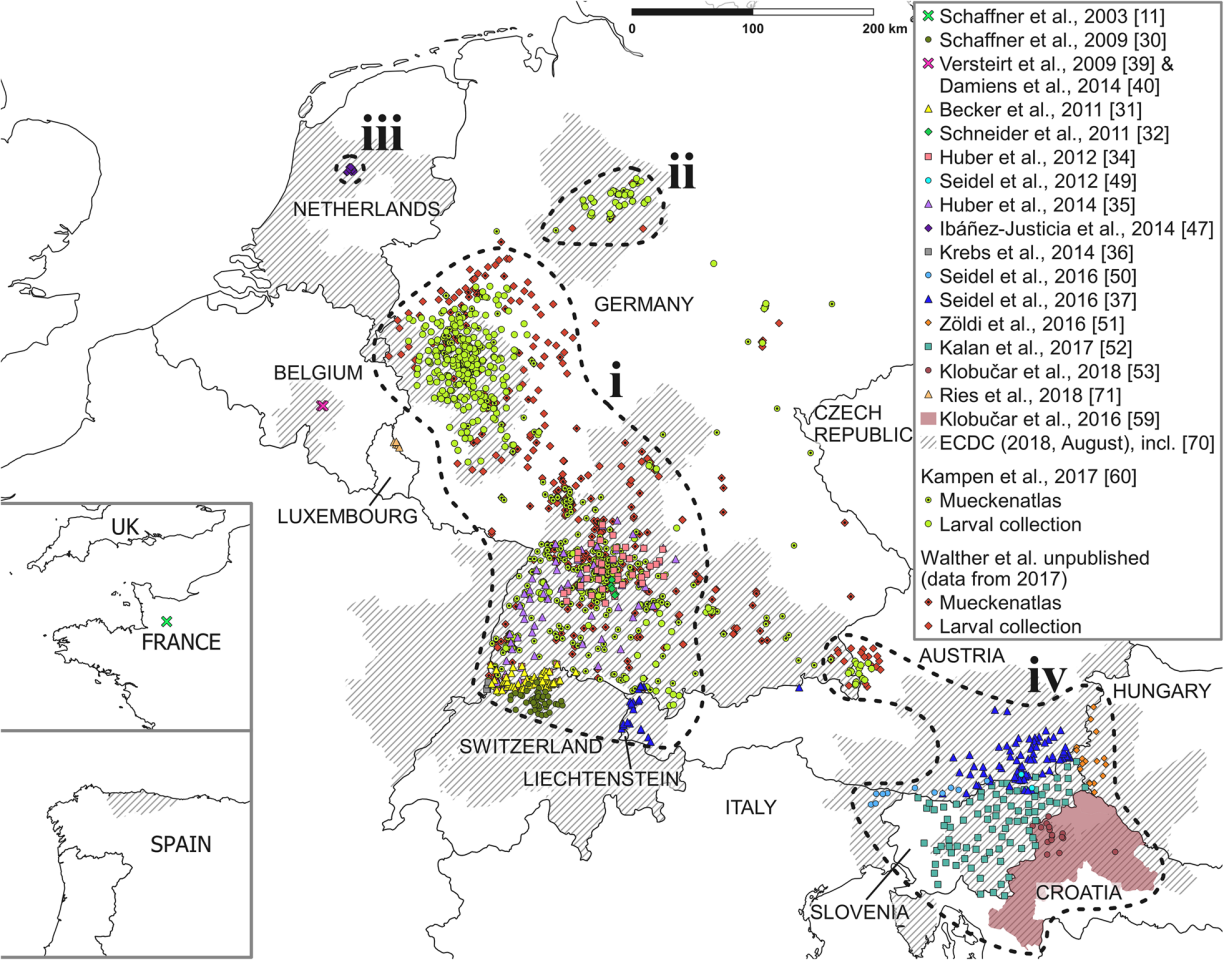
*Aedes j. japonicus* occurrence reported in Europe according to studies published until the end of 2017 plus two online reports from 2018 (coloured symbols; crosses indicate reports of eradication). Dashed outlines and their respective numbers refer to the present populations as mentioned in section “Summary of the European distribution as of 2017”. *Aedes j. japonicus* findings between 2012 and 2016 from studies by Kampen et al. [6, 42, 44], Walther et al. [43], Werner et al. [33, 45] and Zielke et al. [54] are summarised in Kampen et al. [60]; unpublished data from 2017 are referred to as ‘Walther et al., unpublished’. Hatched areas display *Ae. j. japonicus* reporting (introduction and establishment) based on NUTS3 level, according to the ECDC *Ae. j. japonicus* vector map from August 2018 (https://ecdc.europa.eu/en/publications-data/aedes-japonicus-current-known-distribution-august-2018). Hatched areas may be misleading considering the size of the putative distribution areas but are particularly important for following-up affected areas when no recent original data are available, e.g. for Switzerland. The map was created with QGIS, v.2.18.14 (QGIS Development Team, 2018). The base map of Europe and respective administrative areas were downloaded from http://www.naturalearthdata.com

Data collected in Germany in 2016 and 2017 indicate that *Ae. j. japonicus* continued to expand eastwards from the federal state of Baden-Wuerttemberg into the federal state of Bavaria, and northwards into the federal states of Rhineland-Palatinate and Hesse (Fig. 1). In addition, *Ae. j. japonicus* was found in the southeastern part of Rhineland-Palatinate in 2017, close to the German federal state of Saarland (Fig. 1), suggesting that a far larger than known region of French Alsace might be colonised. Unfortunately, except for one finding by Seidel et al. [37], no *Ae. j. japonicus* distribution data have been published from Switzerland after 2009 [30] although the spread has continued, preventing precise mapping (Fig. 1).

### Belgium

In 2002, a surveillance programme targeting *Ae. albopictus* in France [38] led to the first detection of *Ae. j. japonicus* in Belgium. Potential larval breeding sites examined on and around the premises of a tyre-trading company in 2002, 2003, 2004, 2007 and 2008, demonstrated its establishment. A second company ground in the vicinity of the survey area was found to be infested in 2008. Surprisingly, there was no evidence of this population spreading to the surrounding area [39].

The lack of dispersal was confirmed by a follow-up study conducted in 2009. Damiens et al. [40] repeatedly visited the previously affected premises and reported the finding of several larvae on the companies’ grounds while only one larva was found in a puddle 100 m away. Control was initiated in 2012, based on larvicidal Bti (*Bacillus thuringiensis israelensis*)-toxin application and reduction of potential breeding habitats. The Belgian population has been considered eliminated since 2015 [41] but reinvasion from Germany seems probable.

### Western Germany

A third European *Ae. j. japonicus* population was detected in western Germany in 2012 [42], after specimens had been submitted for identification to the German ‘Mueckenatlas’ passive surveillance scheme, a citizen science project [43]. The collection sites and their surroundings, e.g. the gardens of the submitters, were searched for potential breeding sites and developmental stages. After it had become clear that a larger area was colonised, the surveillance was expanded to cemeteries in the villages surrounding the positive localities. Finally, a colonised area of about 2000 km^2^ was found. Three possible origins of this second German population were discussed: (i) a northward spread of the southwestern population; (ii) an eastward spread of the Belgian population; and (iii) an additional introduction. So far, no clarification was possible.

Since its detection, Kampen et al. [44] annually monitored the geographic expansion of this population and found a tremendous increase of the colonised area in 2015. The spread continued in all directions in 2016 and 2017, with branches of the population reaching into central Germany and to, possibly across, the Belgian border in the west (Fig. 1). Additionally, a further expansion to the south took place, which, together with the northward spread of the southwest German population, resulted in the merging of these two populations in 2017 (Fig. 1).

### Northern Germany

In late summer 2012, an *Ae. j. japonicus* female was submitted to the ‘Mueckenatlas’ scheme from northern Germany [45]. Due to some delay in processing and the end of the mosquito season approaching, this case was only followed up in 2013. The survey produced *Ae. j. japonicus* larvae in 25 of 129 monitored cemeteries [45]. It was noted that the infested cemeteries seemed to concentrate along two motorways, indicating that this new population could be an offshoot of the West German population that arose through passive displacement of specimens by cars [45]. This was later confirmed by genetic analyses [46]. During the following years, Kampen et al. [44] continued the survey and showed that, contrary to the West German population, the infested area in northern Germany did not expand but even appeared to have decreased by 30% in terms of area coverage until 2015. Such a decrease could not be confirmed in 2016 and 2017 when additional findings rather suggested stagnancy, with some annual fluctuations (Walther et al., unpublished).

### The Netherlands

In 2012, a female *Ae. j. japonicus* specimen was collected in the municipality of Lelystad [47], the Netherlands, leading to intensified monitoring efforts. In 2013, several adult females were trapped at almost the same site where the specimen had been collected in 2012 and a female was caught in the vicinity of a tyre-trading company. After a larva had been found some 7 km from the first location, the survey area was expanded to the whole municipality in late 2013. The extension brought forth *Ae. j. japonicus* eggs, larvae, pupae and adults in allotment gardens, forested areas and the cemetery of Lelystad. It was not possible to identify the point of entry to the Netherlands [47]. In 2016, control was initiated using source reduction and application of Bti-toxin. The population could be reduced but not eliminated [48].

### Eastern Austria/Slovenia/Hungary/Croatia/Italy

For southeastern Austria and northeastern Slovenia, *Ae. j. japonicus* was recorded for the first time in 2011, when larvae were found some 50–60 km apart [49]. Successive investigations of suitable breeding sites, in human settlements or forests, taking place in 2011 and 2012, demonstrated a large area of infestation in southeastern Austria [37]. Seidel et al. [50] assumed that a westward expansion was quite likely and hypothesised that *Ae. j. japonicus* might soon cross the border to Italy. The area was monitored during the following years, starting in 2013, and *Ae. j. japonicus* was indeed found to have expanded westwards and southwards into North Italy between 2013 and 2015, representing its first detection in Italy [50].

Additionally, Seidel et al. [37] reported an expansion of the Asian bush mosquito to the east. In the summer of 2012, *Ae. j. japonicus* larvae were detected in Hungary, depicting the first detection of an invasive mosquito species in this country. Further specimens were identified in Hungary in 2014 and 2015 along the border to Austria [51].

During the first large-scale study targeting invasive mosquitoes in Slovenia, the whole national territory was surveyed to determine the distribution of *Ae. j. japonicus*, among other invasive species. In total, Kalan et al. [52] monitored 141 municipalities throughout Slovenia in 2013 and 2015, with emphasis on municipalities along major traffic axes. The results showed that since the first detection of *Ae. j. japonicus* close to the Austrian border in 2011 [49], the population had spread over most of northeastern Slovenia. By 2015, *Ae. j. japonicus* was present throughout Slovenia except for a small strip of land adjacent to the border to Italy [52].

In Croatia, Klobućar et al. [53] monitored *Ae. j. japonicus* from 2013 to 2015. In 2013, several *Ae. j. japonicus* larvae were collected in a cemetery, while in 2015, 12 of 369 water-filled vases inspected in four cemeteries were inhabited by *Ae. j. japonicus*. In the city of Zagreb, the species was found for the first time as larvae in a wooden container in 2015. A further spread to several northwestern counties was shown by 2016 [53].

### Southeastern Germany/Austria (federal state of Salzburg)

In 2015, Zielke et al. [54] found another *Aedes j. japonicus* population spanning from southeastern Germany across the border into Austria. According to data collected in 2016 and 2017, this population has since also expanded considerably, both into Germany and into Austria (Fig. 1). As of 2017, the typical character of a population, isolation, was no longer given on the German side as a loose corridor of *Ae. j. japonicus* larval collection sites connected this population with that of southwest Germany (Fig. 1).

### Summary of the European distribution as of 2017

In summary, of the seven populations of *Ae. j. japonicus* that came to attention in Europe, only four still exist as of 2017 due to the elimination of one population (Belgium) and the merging of three others (Fig. 1):

(i) The largest population covered western Germany (parts of the federal states of North Rhine-Westphalia, Rhineland-Palatinate and Hesse), the whole federal state of Baden-Wuerttemberg, from where it crossed the border to France (Alsace) in the west and to Bavaria in the east, interlinking with the southeast German/Austrian (Salzburg) population, and a significant part of northern Switzerland from where it extends to the east through Liechtenstein into western Austria. Regionally, considerable population densities occur.
(ii) A relatively small population which had not spread since its detection in 2013 exists in north Germany in parts of the federal states of Lower Saxony and North Rhine-Westphalia. Due to the ongoing expansion of the West German population to the north, it is expected that both populations will merge in the near future.
(iii) The very small Dutch population remained restricted to the municipality of Lelystad.
(iv) Probably the second largest, the population covered southeastern Austria, northern Italy, almost the whole of Slovenia (except for the most western part) and parts of Croatia and Hungary.

In addition to the *Ae. j. japonicus* reports from Germany allocated to the various populations and federal states, there are scattered findings from the central part of the country (northern Bavaria, Thuringia, Saxony and Saxony-Anhalt) (Fig. 1).

## Genetic relationship of European *Ae. j. japonicus* populations

Population genetic studies support the assumption that at least two separate introductions of *Ae. j. japonicus* mosquitoes into Europe took place since 2000 [35, 55], when the first evidence of *Ae. j. japonicus* was reported from France [11]. Individuals from the Belgian population collected in 2008 and 2012 and subjected to microsatellite analysis rather resembled the subsequently found German/Swiss population than the populations from western and northern Germany regarding their genetic makeup [55].

Cluster analyses based on microsatellite data from all European populations detected until 2015 (except samples from France [36]) clearly show two genotypes of *Ae. j. japonicus* [35, 46, 55]. One cluster, called ‘genotype 1’, includes samples from the early detected populations in Belgium and southwestern Germany/Switzerland as well as from those from eastern Austria/Slovenia and southeastern Germany/northwestern Austria [46, 54, 55]. By contrast, mosquitoes from western and northern Germany represent ‘genotype 2’ [46, 55]. The quite admixed Dutch population shows both genotypes and is thus most likely based on at least two introductions of mosquitoes from different parts of Europe or from overseas [46]. Specimens from the distribution areas in northern and western Germany also share one *nad*4 (NADH dehydrogenase subunit 4 gene) mitochondrial haplotype (“H5”) which was unique in Europe at the time of examination, underlining the assumed origin from different source populations [55]. This haplotype was also detected in Slovenian mosquitoes [55].

The Belgian population is the only one among the European populations for which the introduction pathway could be hypothesised. Individuals were only found on the premises and in the close vicinity of one intercontinentally operating used tyre-trading company [39], indicating its introduction by the international tyre-trade. As for the other European populations, means of introduction and transportation are quite obscure. Active migration of the mosquitoes certainly plays a minor role over long distances [56]. It must be assumed that in some cases (e.g. western and northern Germany, Slovenia and southeastern Germany), motorways, connecting the distribution areas, serve as routes of passive transportation of all life stages [45, 54, 56, 57].

## Methodological appraisal

**Fig. 2:**
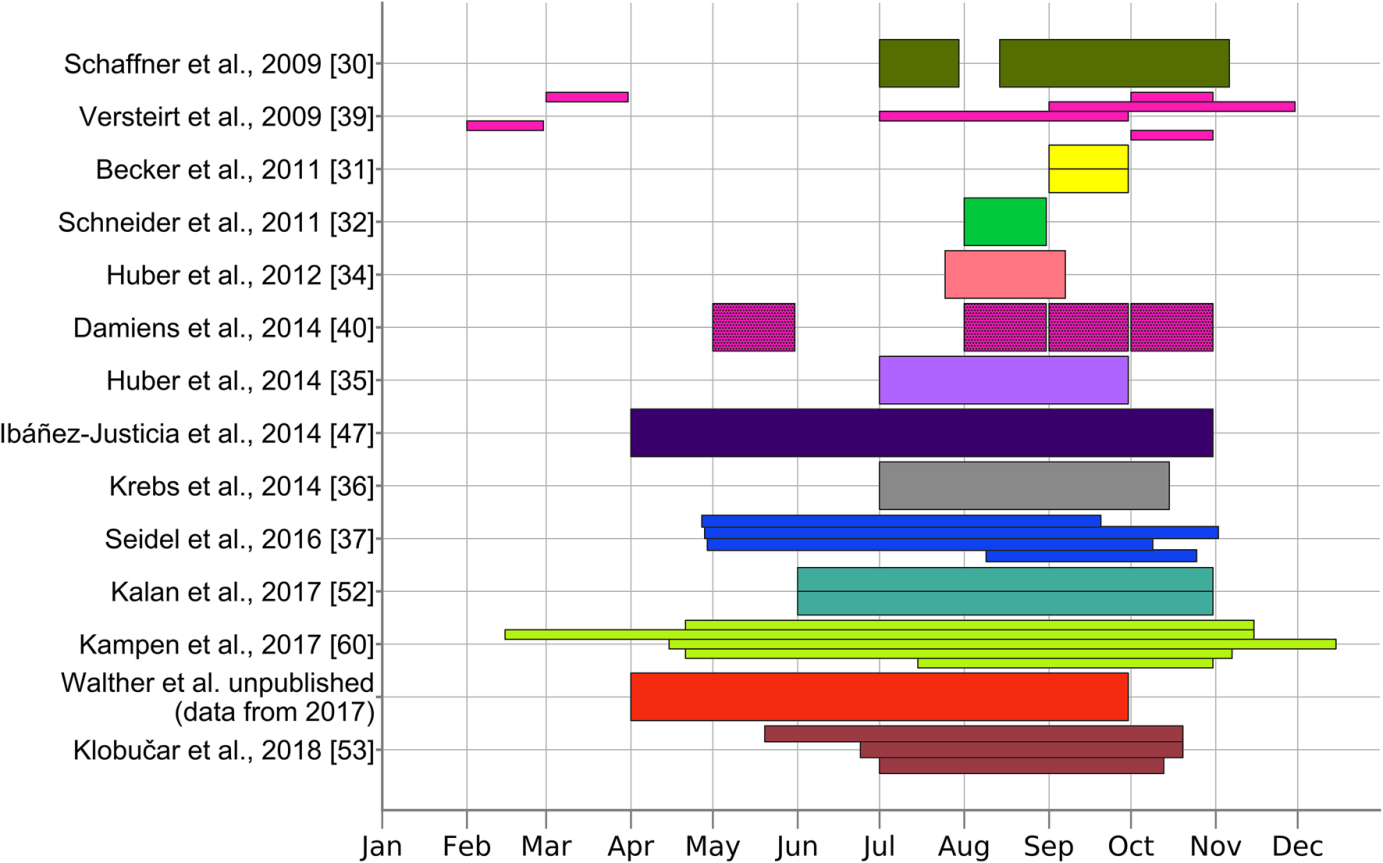
Timelines showing the seasonal periods in which monitoring was carried out. Studies describing monitoring efforts over two or more years are shown with multiple, horizontally separated bars. Studies with imprecisely defined monitoring periods are not included [11, 50, 51]. Graphs were generated with Matplotlib v.2.1.0 for Python v.3.6.4

The review by Vezzani [58], who stressed the importance of cemeteries as highly suitable and readily accepted breeding habitats for container-breeding mosquitoes, considerably influenced the methodology of invasive mosquito surveillance in recent years. This is particularly obvious in large-scale studies. Almost all published surveillance activities used cemeteries, alone or in combination with other landscape structures, e.g. allotment gardens, to monitor infestation and delimit areas inhabited by *Ae. j. japonicus*. Furthermore, most studies are similar considering the season in which the surveillance work was conducted (Fig. 2).

All reviewed studies (except [33]) deal with immature stages, and only few groups used additional adult or oviposition traps. Unfortunately, many reviewed studies are short or rapid communications, thus missing detailed methodological descriptions. For two studies [51, 59], only findings are presented, as the available sources (abstracts from conference talks) provide insufficient methodological information.

Furthermore, the reviewed studies exhibit crucial differences in methodologies applied and details of presentation. First of all, type and quantity of examined containers frequently remain unmentioned. In addition, recurring visits in the same year have been done almost exclusively in the scope of small-scale studies (except for the large-scale study by Kalan et al. [52]), which is likely attributable to the workload. In some cases, the number of visits is not specified. Most importantly, the criteria regarding the declaration of negative sites differ significantly and must be questioned in some cases. While Schaffner et al. [30] used the exclusive presence of culicid larvae other than *Ae. j. japonicus* in one container or the complete lack of culicids in five containers as a negative indicator, other groups used the complete absence of *Ae. j. japonicus* larvae after a certain number of containers checked as an indicator. Furthermore, the number of containers or sites which were screened and found negative differs between the various approaches.

## Recommendations to harmonise monitoring

To standardise and harmonise large-scale surveillance activities (when the species is already found established in an area larger than 25 km^2^), we suggest a halo approach, creating a circle of negative sites around positive sites to define the boundaries of a population, in combination with the use of a grid cell pattern. The principle of this method is based on the studies by Schaffner et al. [30], Huber et al. [34], Kampen et al. [42, 44], Werner & Kampen [45] and Zielke et al. [54]. As a first step, a virtual grid with a defined cell size is generated, as done by Huber et al. [34], Kampen et al. [44] and Zielke et al. [54]. To our knowledge, there are no studies comparing the effect of different grid resolutions; thus, specifying an evidence-based cell size is not feasible. Studies reviewed in this paper either used a cell size of 11 × 12.5 km = 137.5 km^2^ [34] or 10 × 10 km = 100 km^2^ [44, 54, 60], while the ECDC ‘Guidelines for the surveillance of invasive mosquitoes in Europe’ [29] suggest to inspect 40 containers in an area with a maximum size of 25 km^2^ at the very beginning of the colonisation phase. The latter depicts a small-scale approach and would, if projected on a large scale, correspond to 160 containers per 100 km^2^ and 220 containers per 137.5 km^2^.

As mentioned earlier, the review by Vezzani [58] strongly pushed the selection of study sites towards cemeteries. Yet, allotment gardens were also shown to be suitable [48]. We therefore suggest that the search for developmental stages be performed in cemeteries or allotment gardens, owing to several advantages such as time efficiency, a high density of potential breeding sites and a high acceptance by *Ae. j. japonicus* and other invasive *Aedes* species [48, 58]. Cemeteries offer the additional advantage of being public property and therefore easily accessible, which usually is not the case with allotment gardens as these are private property. If neither suitable cemeteries nor allotment gardens can be found or accessed, alternative structures to be searched for developmental stages may include used tyre storages, farms and other locations where small water-holding containers can be found. If possible, the surveyed structures should be located in the vicinity of forested areas, as several studies indicate that *Ae. j. japonicus* uses forest edges to spread [61, 62].

Unless *Ae. j. japonicus* stages are quickly found, all water containers in a suitable structure should be inspected when the structure is small or has a low number of water containers available. In huge structures with a high number of water containers, a pre-determined time limit for inspection (e.g. one hour per location) or number of water containers to be inspected ensures a time-efficient compromise. Huber et al. [34], for example, inspected a minimum number of 30 containers per cell, while Kampen et al. [44] checked at least 80 containers per cemetery if one hour was not enough to inspect all containers. We propose to inspect a minimum of 150 containers per 100 km^2^, which approximately corresponds to the above ECDC recommendations [29]. If available, at least three structures per cell should be inspected, which are to be selected in a way that the imaginary triangular area between them covers an area as large as possible. Especially in rural and mountainous areas it may happen, however, that a cell contains only one or two of the desired structures. In this case, all water bodies in the structures should be searched, ignoring a time limit to compensate for the loss of area coverage. Alternatively, deciduous forests (with tree-holes instead of artificial water containers) can be screened. A grid cell should be rated positive as soon as one single larva or pupa is unambiguously identified. It should be rated negative if no *Ae. j. japonicus* immature stages are found following the aforementioned criteria.

As realised in most of the reviewed studies (Fig. 2), the survey should be conducted during the seasonal activity peak of mosquitoes (August to September), when optimal developmental conditions are provided. In this case, a single areal inspection per year is considered sufficient. Although *Ae. j. japonicus* has been shown to be active from early spring until late autumn [27], population densities might be extremely low in a situation of initial colonisation and cause false negative results outside the activity optimum.

If the density of a population was high (numerous water containers colonised with plenty of larvae) in a previous survey and the workload needed for surveillance turns out to increase significantly, due to a continuous expansion of the population, consecutive monitoring efforts may start with the outermost positive cells of the previous study, assuming that by verifying infestation in the margins of the colonised area determined before, the centre of that area is also still infested. If the border area cell checked first is found negative, the cell adjacent in the direction to the centre of the previously colonised area should be examined before continuing away from the centre.

The suggested approach needs relatively little preparation time as only GIS-software is needed to generate a grid overlay. Furthermore, cemeteries, allotment gardens and land use (e.g. forested areas) can be found as features on open-source GIS layers (e.g. OSM data). Alternatively, web searches can help in detecting suitable locations. Additionally, the costs of this approach are manageable, as the biggest cost factor is travel expenses, and only basic equipment such as small sieves, dippers, pipettes and sample containers are required. Other cost factors, such as training and labour need to be taken in account, although these are always incurred, independent of the applied monitoring technique.

Furthermore, the resulting distribution map is easily understandable (c.f. [44]), hence usable to quickly inform the broader scientific community, and easily comparable regarding the colonised area between different monitoring years and different monitoring regions. Relative to the size of the survey area, the required workforce is low. Two researchers experienced in identifying *Ae. j. japonicus* larvae under field conditions, were able to trace the area of the western Germany population in 2016 within a time period of two weeks, by starting with the outermost positive sites of the distribution area (approx. 8900 km^2^) as found in 2015 [44].

A gradual adoption of the suggested method in surveying *Ae. j. japonicus* in the years to come may pave the way for large-scale collaborations regarding data analysis and the design of predictive models, while the standardisation will generally support future mosquito research. However, collecting data is only one part of large-scale cooperation, as it could be beneficial to establish a database which explicitly contains presence and absence data. Our proposed method could also prove useful for other invasive *Aedes* species, since no standardised approach for large-scale larval surveillance can be found in the pertinent European literature for either *Ae. albopictus* [63–67] or *Ae. koreicus* [68, 69].

## Conclusions

By late 2017, 17 years after the first detection of *Ae. j. japonicus* in Europe, this invasive mosquito species was demonstrated to be established in ten countries, in most of which it continues to spread. Only in Belgium could it be eliminated after several years of restricted local occurrence. In June and July 2018, *Aedes j. japonicus* was detected for the first time in northern Spain [70] and Luxembourg [71], respectively, increasing the number of infested European countries to 12. As these two recent reports were short online notifications without any details, they are mentioned here for completeness only (Fig. 1). Even if eradication of *Ae. j. japonicus* no longer seems feasible, further surveillance may add important information for mosquito-borne disease risk assessments and is an opportunity to study the spread and the occupation of an ecological niche by a newly emerging climatically adapted species. Additionally, monitoring data of several years could reveal environmental conditions, such as specific landscape structures, which support or impede the spread. To improve future research and harmonisation of data collection, we propose a methodological approach for the continuous surveillance of populations which infest an area larger than 25 km^2^ (c.f. [29]), that pose a high risk of further spread. This methodological approach could be further used as a framework for more detailed data collections including key figures, such as container indices, larval counts, occurrence of species-coexistence, exact counts of positive containers per container type, environmental data, etc. Recording such detailed data will of course require more time and increase costs both directly (labour and training) and indirectly (possible additional tools), yet it could prove useful for meta-studies and modelling approaches, as most existent studies only work with presence/absence data and climate data [52, 72–75]. The use of other strategies, e.g. trapping of eggs and adults, can be suitable in situations where the efforts aim to determine initial establishment, population density, small-scale distribution or presence of pathogens (e.g. [23, 24, 45]). Utilisation of stationary traps in large-scale surveys (e.g. [60]) does not seem feasible, or will at least prove expensive and be linked to a wide range of problems, e.g. malfunctions, theft or demolition. Ovitrap networks are also cost-efficient but increase travel expenses as at least two visits per site are needed (setup and removal) and usually do not allow species identification on the spot.
